# Role of Cardiomyocyte-Derived Exosomal MicroRNA-146a-5p in Macrophage Polarization and Activation

**DOI:** 10.1155/2022/2948578

**Published:** 2022-05-02

**Authors:** Cong Chen, Sidong Cai, Min Wu, Run Wang, Mingya Liu, Gaozhen Cao, Min Dong, Kai-Hang Yiu

**Affiliations:** ^1^Division of Cardiology, Department of Medicine, The University of Hong Kong Shenzhen Hospital, Shenzhen, China; ^2^Bioland Laboratory (Guangzhou Regenerative Medicine and Health Guangdong Laboratory), China

## Abstract

Myocardial infarction arises from an excessive or prolonged inflammatory response, leading to ventricular remodeling or impaired cardiac function. Macrophages exhibit different polarization types associated with inflammation both at steady state and after myocardial infarction. Exosomal miR-146a-5p has been identified as an important molecule in the cardiovascular field in recent years. However, the effect of cardiomyocyte-derived exosomal miR-146a-5p on macrophages has not yet been elucidated. Initially, we found that exosomes with low expression of miR-146a-5p derived from myocardial infarction tissues modulated macrophage polarization. To determine whether cardiomyocyte-derived exosomal miR-146a-5p mediated macrophage polarization, we treated macrophages with exosomes rich in miR-146a-5p collected from neonatal mouse cardiomyocytes. The effects of exosomal miR-146a-5p on macrophage polarization were measured using RT-qPCR, transwell assays, and western blotting. The results showed that the increased expression of miR-146a-5p promoted M1 macrophage polarization, inhibited M2 macrophage polarization, and increased the expression of VEGFA. However, the decreased expression of exosomalmiR-146a-5p showed the opposite trends. Interestingly, in contrast to treatment with the solitary miR-146a-5p mimic, exosomal miR-146a-5p derived from neonatal mouse cardiomyocytes reduced TNF*α* and iNOS expression. In addition, when macrophages were activated by the miR-146a-5p mimic or exosomal miR-146a-5p, the expression of TNF receptor-associated factor 6 (TRAF6), a target gene of miR-146a-5p, was reduced significantly. Taken together, these findings indicate that exosomal miR-146a-5p derived from cardiomyocytes could stimulate M1 macrophage polarization to induce an inflammatory reaction, while targeting TRAF6, exerting an anti-inflammatory effect. Exosomal miR-146a-5p plays important roles in macrophages, illuminating a novel potential therapeutic target in myocardial infarction.

## 1. Introduction

Over the past decade, the prognosis of myocardial infarction (MI) has improved significantly, and MI remains the main cause of morbidity and mortality worldwide [1]. In most cases, myocardial infarction is due to rupture of a fragile atherosclerotic plaque, and these vulnerable plaques contain more inflammatory cells such as macrophages [1, 2]. In human and experimental animal models, macrophages infiltrate the injured area after myocardial infarction [3]. As important natural immune cells, macrophages play an important role in the inflammatory response and cardiac remodeling after myocardial infarction through phagocytosis of necrotic cells and debris [4]. Macrophages accumulate lipids and secrete cytokines, which can attract other leukocytes, produce proteases, digest extracellular matrix, interfere with smooth muscle cell function, and affect endothelium-dependent vasodilation [5].In particular, a substantial amount of research demonstrates the therapeutic potential of macrophages as targets for nanoparticle-mediated drug delivery systems [6]. Therefore, it is significant to explore the role of macrophage activation and its potential as a treatment for myocardial infarction.

Cardiosphere-derived cell-secreted exosomal microRNAs (miRNAs) play an important role in macrophage polarizations [7]. Studies have found that miRNAs derived from small extracellular vesicles from mesenchymal stem cells can promote angiogenesis to repair myocardial injury [8]. MiRNAs can participate in cellular communication through exosomes [9].Exosomes originate from different subcellular regions and are released into the extracellular space. By transferring their cargo to target cells and tissues, exosomes act as novel regulators of intercellular communication between adjacent and distal cells [10].Because the composition and biological inclusions of exosomes exhibits specific characteristics of cell activation and injury, their potential as diagnostic and prognostic biomarkers have aroused great interest in cardiovascular disease. Pericardial fluid exosomes are rich in cardiovascular miRNAs, which can promote angiogenesis [11]. Studies have found that the expression of miR-146a-5p was decreased significantly in patients with ST elevation myocardial infarction (STEMI) compared to control group patients [12]. TNF receptor-associated factor 6 (TRAF6) is an essential molecule of proinflammatory pathways via activation of NF-*κ*B [13]. Downstream of TRAF6, the phosphorylation of inhibitor of NF-*κ*B kinase *α*, and *β*(IKK*α*/*β*) plays a crucial role in the activation of the transcription factor NF-*κ*B [14]. TRAF6 is a direct target of miR-146a-5p [15]. Meanwhile, studies had found that hyper-IL-6 induced the overexpression of miR-146a-5p [16]. miR-146a-5p participates in anti-inflammatory or proinflammatory processes involving the NF-*κ*B signaling pathway [17–19].

The mechanism by which cardiomyocyte-derived exosomal miR-146a-5p modulates macrophage populations, however, is currently unknown. In this study, we studied the effect of exosomal miR-146a-5p on macrophage differentiation and polarization to uncover the mechanism behind macrophage modulation. These results provide insights into the mechanism of cardiomyocyte-derived exosomes involved in macrophage polarization for myocardial infarction.

## 2. Materials and Methods

### 2.1. Exosome Isolation and Characterization

Plasma samples were collected from 5 healthy individuals and 5 myocardial infarction patients. Patients and individuals' information is shown in Table 1. The plasma was centrifuged at 500 g for 5 min, and the collected supernatant was again centrifuged at 12000 × g, 4°C for 10 min, and stored at −80°C. Both the patients and healthy volunteers consented for sample collection and molecular testing, and the project was approved by the University of Hong Kong-Shenzhen Hospital Research Ethics Committee. All the studies were conducted in accordance with the Helsinki Declaration (1964), and the experiments were conducted with the human subjects' understanding and consent. Exosomes were isolated from 250 *μ*l of plasma using a System Bioscience (SBI) ExoQuick™ Exosome Precipitation Kit. Exosome size and characterization were made using transmission electron microscopy (TEM). A copper mesh was placed on a clean wax plate, and 10 *μ*l of the exosome suspension was added for 5 min. The copper mesh was removed, and 2% phosphotungstic acid was placed on the mesh for 5 min. The mesh was placed on filter paper, and TEM was used to observe the morphological features of the exosomes. Nanoparticle tracking analysis (NTA, Particle Metrix, Germany) was used to determine the particle diameter of the exosomes [20]. The NTA software ZetaView 8.04.02 SP2 (Particle Metrix, Germany) was used for data acquisition and processing according to the manufacturer's instructions. The exosomes were diluted 1 : 50 with vesicle-free DPBS, and the pH was adjusted to 7.0. The ambient temperature was set at 24°C, whereas background extraction and automatic settings were applied for the minimum expected particle size, minimum track length, and blur.

### 2.2. Cell Culture

RAW264.7 macrophage cells were cultured in DMEM (Gibco, USA) supplemented with 10% (v/v) serum replacement (Gibco, USA), 2 *μ*ml-glutamine (Beyotime, China), 100 *μ*g/ml streptomycin, and 100 U/ml penicillin.

### 2.3. Primary Cardiomyocytes of Neonatal Mice

All animal protocols used in this study were reviewed and approved by the Institutional Animal Care and Use Committee at Shenzhen University. Cardiomyocytes were isolated and cultured using previously described methods [21]. Briefly, cardiomyocytes of neonatal mice were isolated from 2 to 3 day-old mice. Isolated cardiomyocytes were plated on 0.1% gelatin solution-coated plates in minimal essential medium *α* with 10% fetal bovine serum and then 24 hours postseeding, the medium was replaced with Dulbecco's Modified Eagle Medium with 2% FBS and 25 *μ*g/ml gentamicin.

### 2.4. Immunocytochemistry

To label exosomes with PKH26 and cells with PKH67, Fluorescent Cell Linker Kits for General Cell Membrane Labelling (Sigma, St. Louis, MO) were purchased and used according to the manufacturer's protocol. In brief, exosomes were resuspended. Then, the resuspended exosomes were stained for PKH26 at a final concentration of 2 M. After 5 min of incubation at 37°C, the reaction was stopped by the addition of 5% BSA. After 1 min of incubation, labeled exosomes were washed three times with PBS and resuspended in PBS. RAW264.7 macrophages were collected, and 2 *μ*l of PKH67 was added. The cells were gently aspirated and mixed well. Fluorescently labeled exosomes and cells were examined under a microscope (OLYMPUS, Japan).

### 2.5. Quantitative Real-Time-PCR Analysis of the mRNA Expression

Total RNA was extracted using the TRIpure Total RNA Extraction Reagent method (ELK Biotechnology, China). RNA concentration and purity were determined using a NanoDrop 2000 (Thermo Fisher Scientific). First-strand cDNAs were synthesized from total RNA using the EntiLink™ 1st Strand cDNA Synthesis Kit (ELK Biotechnology, China) following the manufacturer's instructions. The specific PCR primers used for SYBR Green-based RT-PCR are provided in Table S1. Quantitative real-time-PCR (RT-qPCR) was performed with the Power SYBR Green PCR Master Mix (Thermo Fisher Scientific) to examine the expression levels of markers. The sequences of the primers used are shown in Supplemental file 1: Table S1. All PCR amplifications were performed using a StepOnePlus System (Thermo Fisher Scientific) with an initial denaturation step at 95°C for 3 min, followed by 40 cycles of 9°°C for 15 s, and 58°C for 30 s and a final automatic melting curve stage. Samples were run in triplicate, and the gene expression was determined by 2-*ΔΔ*Ct analysis using GAPDH as a reference.

### 2.6. Detection of miR-146a-5p

Total RNA was extracted using the TRIpure Total RNA Extraction Reagent method (ELK Biotechnology, China). The purified RNAs were analyzed using a Bioanalyser 2100. To confirm the expression of miR-146a-5p, miR-146a-5p was measured using the EntiLink™ 1st Strand cDNA Synthesis Kit (ELK Biotechnology, China) and EnTurbo™ SYBR Green PCR SuperMix (ELK Biotechnology, China). PCR was performed at 42°C for 60 min, 85°C for 5 min, and 40 cycles of amplification at 95°C for 10 s, 58°C for 30 s, and 72°C for 30 s. The melt curve stage was performed at 95°C for 30 s, 60°C for 30 min, and 95°C for 30 s. Relative changes in expression were determined using the 2-*ΔΔ*Ct formula and using U6 served as a reference gene. The sequences of the primers used were as follows: miR-146a-5p, forward, 5′-CCTGAGAAGTGAATTCCATGGG-3′ and reverse, 5′-CTCAACTGGTGTCGTGGAGTC-3′; U6, forward, 5′-CTCGCTTCGGCAGCACAT-3′ and reverse, 5′-AACGCTTCACGAATTTGCGT-3′.

### 2.7. Transient Transfection of miR-146a-5p Mimic and miR-146a-5p Inhibitor

miR-146a-5p mimic, miR-146a-5p inhibitor, or miRNA mimic and inhibitor negative control oligonucleotide consisting of a random sequence of bases (RIBOBIO, China) were transiently transfected into cells by using Lipofectamine 2000 (Invitrogen, Carlsbad, CA, USA) in accordance with the manufacturer's instructions. In brief, transfection was performed with 3 × 10^5^ cells/well in a 6-well plate. The mimic, inhibitor, and negative control oligonucleotide at a final concentration of 100 nm were added to each well. After 6 hours, Opti-MEM was removed and replaced with DMEM. The cells were collected after continuous culture for 24 hours.

### 2.8. Western Blotting

Whole-cell lysates were prepared in radioimmunoprecipitation assay (RIPA) buffer (ASPEN Biotechnology, China) for immunoblotting. Protein concentrations were determined using the BCA Protein Assay Kit (Thermo Fisher Scientific Inc.), according to the manufacturer's instructions, and bovine serum albumin served as the standard. Samples were resolved via sodium dodecyl sulfate–polyacrylamide gel electrophoresis under reducing conditions and transferred to polyvinylidene difluoride membranes (Millipore, USA). Target bands were visualized using a Tanon-5200 Image Analyser. Protein band intensity was quantified via densitometry using ImageJ 1.48 software (National Institutes of Health, Bethesda, MD, USA). Primary antibodies and secondary antibodies were purchased from Abcam and Cell Signaling Technology. The information and dilution concentrations of the primary antibodies and secondary antibodies are shown in Table 2.Target bands were visualized using a Tanon-5200 Image Analyser. Protein band intensity was quantified via densitometry using ImageJ 1.48 software (National Institutes of Health, Bethesda, MD, USA).

### 2.9. Transwell Migration Assay

A Transwell system (Corning, New York, NY, USA) with an 8 *μ*m-pore filter was used to evaluate the migration potential of the derived cells. Briefly, 5 × 10^4^ cells in serum-free medium were seeded into the upper chamber of the insert, and complete medium was added to the lower chamber. Cells were incubated for 24 h at 37°C in a 5% CO_2_ incubator. Nonmigrating cells were removed from the top surface of the filter by using a cotton swab, whereas the migrating cells that traversed to the bottom chamber membrane were fixed with 4% paraformaldehyde for 10 min. After fixation, membranes were cut using a blade and stained with DAPI (Life Technologies, USA). The number of migrated cells was counted in five random fields per filter and observed at ×100 magnification under an inverted fluorescence microscope (OLYMPUS, Japan).

### 2.10. Statistical Analysis

Data are presented as the mean ± SEM. Two-tailed independent Student's *t*-test was used to statistically analyze the data. *P* < 0.05 was considered statistically significant. A column chart was drawn using the GraphPad Prism (version 6.0) statistical program.

## 3. Results

### 3.1. Role of Exosomes in the Stimulation of Macrophage Polarization

**Figure 1:**
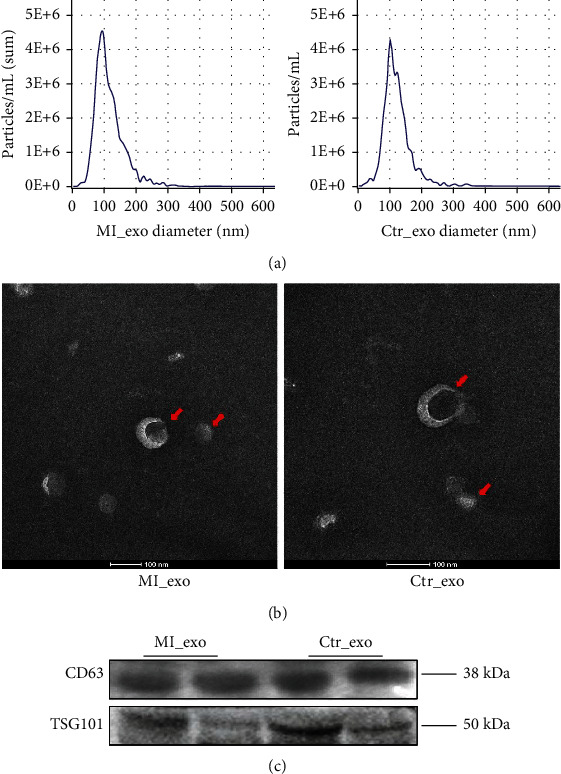
Exosome characteristics. (a) The concentration and diameter of isolated exosomes were detected by NTA. (b) Typical transmission electron microscopy morphology of plasma exosomes. (c) Exosome protein marker CD63 and TSG101 were detected by western blotting in isolated exosomes. MI_exo: exosomes derived from myocardial infarction plasma; Ctr_exo: exosomes derived from healthy individual plasma.

First, exosomes (30–200 nm in diameter) were isolated from the plasma samples of myocardial infarction patients and healthy individuals and detected by NTA (Figure 1(a)). TEM and western blotting were used to detect the diameter and markers of exosomes (Figures 1(b) and 1(c)). These results were consistent with previously reported characteristics of exosomes, confirming that we have successfully purified the exosomes from plasma samples.

**Figure 2:**
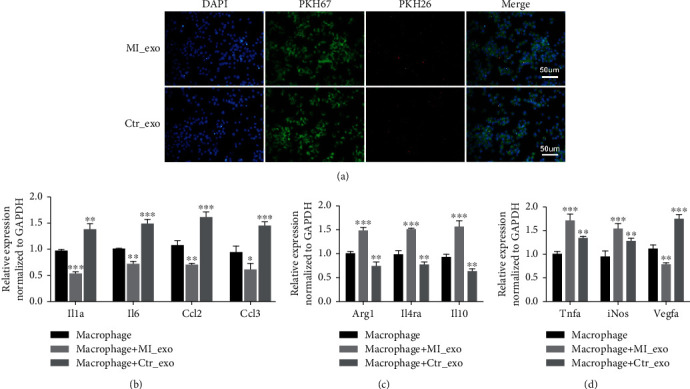
Exosomes derived from patients in myocardial infarction stimulated macrophage polarized activation (a) Double immunostaining showing the phagocytosis of macrophages (PKH67, green) on isolated exosomes (PKH26, red), scale bar = 50 *μ*m.(b)-(d) Relative expression levels of genes related macrophages polarization (^∗^*P* < 0.05, ^∗∗^*P* < 0.01, ^∗∗∗^*P* < 0.001). MI_exo: exosomes derived from myocardial infarction plasma; Ctr_exo: exosomes derived from healthy individual plasma.

To better confirm of macrophage phagocytosis, exosomes from both groups were labeled with PKH26 (red) and incubated with macrophages. Macrophages were stained with PKH67 (green) and imaged with a fluorescence microscope. The results showed that exosomes were phagocytosed by macrophages (Figure 2(a)). RT-qPCR was performed for analysis of genes associated with macrophage M1/M2 polarization. Interestingly, IL-1, IL-6, CCL2, and CCL3 expression was reduced, whereas Arg1, IL4R*α*, and IL-10 expression were increased in macrophages incubated with exosomes derived from the myocardial infarction group (Figures 2(b) and 2(c)). The expression of TNF*α* and iNOS was increased while the expression of VEGFA was reduced in the myocardial infarction group (Figure 2(d)).

**Figure 3:**
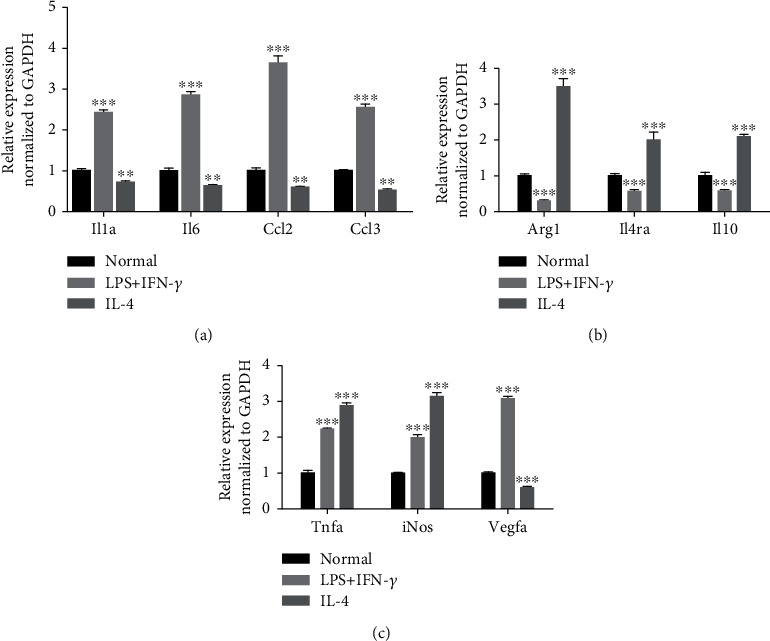
The expression of genes in M1 and M2 polarization. (a) The expressions of IL-1, IL-6, CCL2,and CCL3 genes in M1 and M2 polarization. (b) The expressions of Arg1, IL4R*α*, and IL-10 genes in M1 and M2 polarization. (c) The expressions of iNOS, TNF*α*, and VEGFA genes in M1 and M2 polarization. ^∗^*P* < 0.05, ^∗∗^*P* < 0.01, ^∗∗∗^*P* < 0.001.

To assess macrophage polarizations, we induced M1 macrophage activation of RAW264.7 macrophages by lipopolysaccharide (LPS) and interferon-gamma (IFN-*γ*) incubation for 12 h and M2 macrophage activation via interleukin-4 (IL-4) incubation for 48 h. Compared to the normal group, the expression levels of the IL-1*α*, IL-6, CCL2, and CCL3 genes were increased in M1 macrophages but decreased in M2 macrophages (Figure 3(a)). Compared to the normal group, the expression level of Arg1, IL4R*α*, and IL-10 were decreased in M1 macrophages induced by LPS and IFN-*γ* but increased in M2 macrophages induced by IL-4 (Figure 3(b)). Interestingly, bothM1 and M2 macrophages highly expressed TNF*α* and iNOS (Figure 3(c)). Meanwhile, the expression of VEGFA raised in M1 macrophages induced by LPS and IFN-*γ* (Figure 3(c)). Exosomes derived from myocardial infarction patients may induce macrophage polarization. These results also indicated that exosomes from myocardial infarction may have a potential impact on inflammation and angiogenesis.

### 3.2. miR-146a-5p Modulated Macrophage Polarized Activation

**Figure 4:**
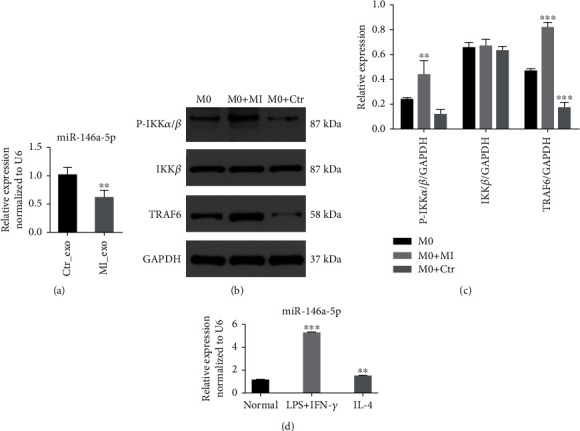
The expression of miR-146a-5p. (a) miR-146a-5p expression levels in RAW264.7 macrophages incubated with different exosomes. (b, c) The protein levels of TRAF6 and phosphorylated IKK*α*/*β* in RAW264.7 macrophages incubated with exosomes derived from MI and healthy individuals. (d) The relative level of miR-146a-5p in M2 and M1 polarization. ^∗^*P* < 0.05, ^∗∗^*P* < 0.01, ^∗∗∗^*P* < 0.001.

Next, miR-146a-5p levels in exosomes derived from the myocardial infarction group were markedly decreased, compared with the healthy individual group (Figure 4(a)). TRAF6 is a target gene for miR-146a and may play a crucial role in inflammation. Therefore, to clarify miRNA-mediated regulation on the TRAF6 expression, TRAF6 protein levels were detected by western blotting. The expression of TRAF6 and phosphorylated IKK*α*/*β* was increased in the group of exosomes from myocardial infarction with low expression of miR-146a-5p compared with the healthy subjects (Figures 4(b) and 4(c)). Moreover, RT-qPCR was performed to measure the expression of miR-146a-5p in M1 and M2 polarized macrophages. The miR-146a-5p expression was upregulated in M1 macrophages compared with in M2 macrophages (Figure 4(d)). Exosomes with decreased miR-146a-5p induced macrophage polarization.

**Figure 5:**
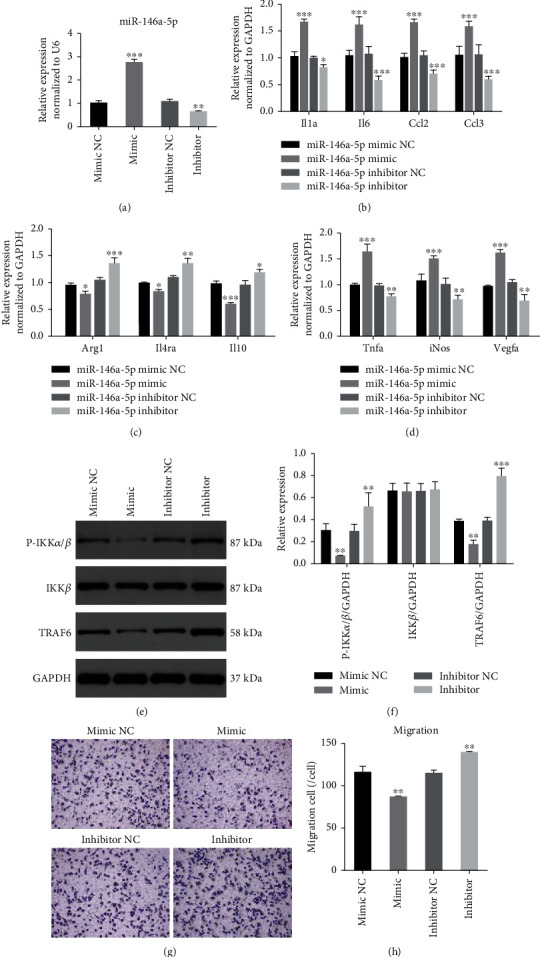
miR-146a-5p modulates macrophage polarization. (a) The expression level of miR-146a-5p after miR-146a-5p mimic, miR-146a-5p inhibitor, miRNA mimic NC, and inhibitor NC transfected in RAW264.7 macrophages. (b)–(d) The expression level of Arg1, IL4RA, IL-10, IL-6, IL-1, CCL2, CCL3, VEGFA, TNF*α*, and iNOS in macrophages with miR-146a-5p mimic, miR-146a-5p inhibitor, miRNA mimic NC, and inhibitor NC treatment. (e)–(f) The protein levels of TRAF6 and phosphorylated IKK*α*/*β* in macrophages with different treatments. (g) Transwell migration assays. (h) Statistics of the number of migrated cells; scale bar = 50 *μ*m. mimic NC: miRNA mimic negative control group; mimic: miR-146a-5p mimic group; inhibitor NC: miRNA inhibitor negative control group; inhibitor: miR-146a-5p inhibitor group. ^∗^*P* < 0.05, ^∗∗^*P* < 0.01, ^∗∗∗^*P* < 0.001.

To further investigate the role of miR-146a-5p in macrophage polarization, RAW264.7 macrophages were treated with miR-146a-5p mimic, miR-146a-5p inhibitor, and their normal controls. miR-146a-5p expression levels were markedly increased in the miR-146a-5p mimic group but decreased in the miR-146a-5p inhibitor group (Figure 5(a)). IL-1*α*, IL-6, CCL2, and CCL3 gene expression was significantly increased in macrophages cultured with the miR-146a-5p mimic but decreased with the miR-146a-5p inhibitor (Figure 5(b)). The levels of the Arg1, IL4R*α*, and IL-10 genes were reduced significantly in macrophages transfected with the miR-146a-5p mimic but increased in macrophages transfected with the miR-146a-5p inhibitor (Figure 5(c)). In addition, iNOS, TNF*α*, and VEGFA levels were increased upon the miR-146a-5p overexpression (Figure 5(d)). These results reveal that overexpressed miR-146a-5p induced M1 polarization, whereas the decreased miR-146a-5p expression stimulated M2 polarization.

Interestingly, the expression of TRAF6 and phosphorylated IKK*α*/*β* was significantly reduced in both RAW264.7 macrophages treated with miR-146a-5p mimic but increased in the group treated with miR-146a-5p inhibitor (Figures 5(e) and 5(f)). Transwell migration assays were used to evaluate the chemotaxis of macrophages. Increased miR-146a-5p levels inhibited migration to attenuate macrophage chemotaxis. Conversely, decreased miR-146a-5p levels promoted macrophage chemotaxis (Figures 5(g) and 5(h)). These results indicated that miR-146a-5p may play a dual role in inflammation.

### 3.3. Role of Exosomal miR-146a-5p as an Anti-Inflammatory and Proinflammatory Agents

**Figure 6:**
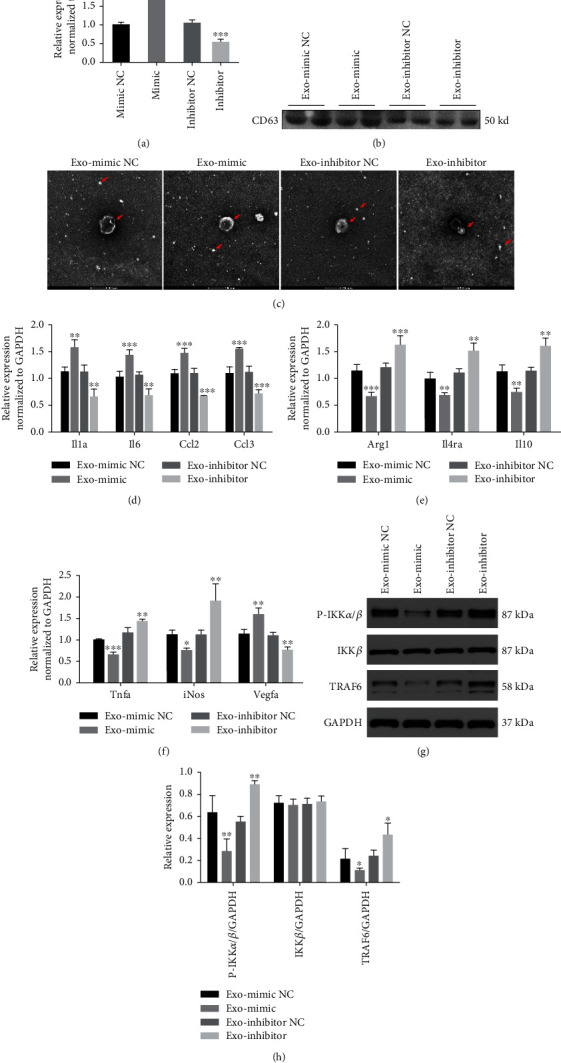
Neonatal mouse cardiomyocyte-derived exosomal miR-146a-5p acted a dual role of anti-inflammatory and pro-inflammatory. (a) The expression level of miR-146a-5p in exosomes derived from cardiomyocytes with miR-146a-5p mimic, miR-146a-5p inhibitor, miRNA mimic NC, and inhibitor NC treatment. (b) Western blot of exosome protein marker CD63 detection. (c) Exosome morphological identification by transmission electron microscopy derived from cardiomyocytes with different treatment. (d)–(f) Relative expression levels of genes in macrophages incubated with different exosomes. (g) and (h) The protein levels of TRAF6 and phosphorylated IKK*α*/*β* in macrophages incubated with different exosomes derived from cardiomyocytes. (^∗^*P* < 0.05, ^∗∗^*P* < 0.01, ^∗∗∗^*P* < 0.001). Exo-mimic NC: exosomes derived from cardiomyocytes treated with the miRNA mimic negative control group; exo-mimic: exosomes derived from cardiomyocytes treated with the miR-146a-5p mimic group; exo-inhibitor NC: exosomes derived from cardiomyocytes treated with the miRNA inhibitor negative control group; exo-inhibitor: exosomes derived from cardiomyocytes treated with the miR-146a-5p inhibitor group.

To determine whether the exosomal miR-146a-5p derived from cardiomyocytes contributes to macrophage polarization, neonatal mouse cardiomyocytes were transfected with miR-146a-5p mimic, miR-146a-5p inhibitor, and their normal controls. RT-qPCR results showed that the miR-146a-5p expression was increased in exosomes treated with miR-146a-5p mimic but decreased in exosomes from cardiomyocytes treated withmiR-146a-5p inhibitor (Figure 6(a)).Initially, western blotting and TEM were used to validate the purified exosomes (Figures 6(b) and 6(c)). Next, these exosomes from different groups of cardiomyocytes were incubated with RAW264.7 macrophages for 24 h. RT-qPCR results showed that IL-1, IL-6, CCL2, and CCL3 expression levels were increased, whereas Arg1, IL4R*α*, and IL-10 expression levels were decreased in macrophages with exosomal miR-146a-5p (Figures 6(d) and 6(e)). Interestingly, the qRT-PCR showed that TNF*α* and iNOS levels were reduced in the macrophage exosomal miR-146a-5p group (Figure 6(f)). The VEGFA expression was increased in the exosomal miR-146a-5p group (Figure 6(f)).When RAW264.7 macrophages were incubated with exosomes derived from cardiomyocytes in different groups, TRAF6 and phosphorylated IKK*α*/*β* protein levels were decreased in macrophages incubated with exosomal miR-146a-5p (Figures 6(g) and 6(h)). These data suggest that miR-146a-5p negatively regulates TRAF6 activation in macrophages and that cardiomyocyte-derived exosomal miR-146a-5p modulates macrophage polarization and may inhibit inflammation in macrophages.

## 4. Discussion

Macrophages are a population of cells with plasticity and pluripotency that show obvious functional differences under the influence of different microenvironments in vivo and in vitro [22]. Macrophages in different tissues differ from each other and have different transcriptional profiles and functions, but all macrophages exhibit a dynamic balance [23]. The transformation of macrophages from a proinflammatory phenotype to reparative phenotype can limit inflammation, contribute to the repair of myocardial infarction, and improve cardiac remodeling and prognosis after MI [24]. Therefore, it is of great significance to identify the endogenous regulatory mechanism of the macrophage functional phenotype for early intervention in myocardial infarction. In this study, we demonstrated that cardiomyocyte-derived exosomal miR-146a-5p could polarize macrophages.

Macrophages can be polarized into the M1 phenotype (classically activated macrophages) and M2 phenotype (alternatively activated macrophages) according to their activation status and function [25].M1 macrophages are involved in the proinflammatory response and play a central role in host defense against bacterial and viral infections [26]. M2 macrophages are associated with the anti-inflammatory response, tissue remodeling, fibrosis, and tumor development [27]. The polarization state of M1 macrophages is driven by IFN-*γ* and LPS, whereas M2 macrophages stimulated with IL-4 comprise a continuum of functional states with involving a large number of factors [28]. Once macrophages are polarized, they still retain the ability to respond to the new environment [29].This phenomenon is called the reversibility of polarization and is also known as functional adaptability. This feature has important therapeutic value [30]. Different phenotypes of macrophages remained differentially expressed markers in various disorders. In the current study, M1 phenotype macrophages secreted cytokines, such as IL-6, IL-1, CCL2, and CCL3, whereas M2 phenotype macrophages secreted cytokines, such as Arg1, IL4R*α*, and IL-10. Our results found that macrophages overexpressing miR-146a-5p could induce M1 polarized macrophages, whereas poor miR-146a-5p altered the polarized phenotype state.

Exosomes contain a variety of biological molecules, such as miRNAs, and the abundance of these exosomal miRNAs is uniquely altered in different environments. Cardiosphere-derived exosomal miR-146a-5p is proangiogenic and cardioprotective. This miRNA inhibits proinflammatory cytokines and might be associated with reduced myocardial fibrosis [31]. Exosomes secreted by human cardiac-resident mesenchymal progenitor cells were highly enriched in miR-146a-5p and reduced iNOS expression [32].Previous studies have shown that the reduced miR-146a-5p expression increases the risk of major adverse cardiovascular events in nonstroke patients [33]. Ding et al. found that extracellular vesicles from M1 macrophages inhibited trophoblast migration and invasion by transferring miR-146a-5p [34]. We found that exosomes from myocardial infarction patients with a low level of miR-146a-5p promote macrophage polarization but induce an increase of TNF*α* and iNOS expression. Increased TNF*α* and iNOS levels associated with heart failure [35, 36]. In our study, exosomal miR-146a-5p from neonatal mouse cardiomyocytes modulated M1 macrophage polarization, whereas it reduced proinflammatory cytokines (TNF*α* and iNOS). Interestingly, in the absence of exosomes, the expression of proinflammatory cytokines (TNF*α* and iNOS) was increased in macrophages transfected with the miR-146a-5p mimic. These results indicate that upon treatment with exosomal miR-146a-5p, macrophages were not simplified to exhibit as inflammatory M1 polarization. Exosomal miR-146a-5p from cardiomyocytes has a potentially important role in myocardial infarction treatment.

Studies have found that exosomes can promote vascular tissue repair after ischemic injury [37]. VEGFA is a major driver of angiogenesis and vasculogenesis [38]. VEGFA promotes angiogenesis after MI by increasing ROS production and enhancing autophagy mediated by infarct endoplasmic reticulum stress [39]. The suppression of miR-146a-5p simultaneously inhibited angiogenesis and the expression of the proangiogenic protein EMMPRIN in tumor cells [40].This study showed that both overexpressed miR-146a-5p and exosomal miR-146a-5p increased VEGFA expression. In summary, exosomal miR-146a-5p not only reduces inflammation but also promotes angiogenesis, demonstrating that it is a good potential therapeutic target for myocardial infarction.

TRAF6 is the target gene of miR-146a-5 [41]. Exosomal miR-146a-5p derived from human umbilical cord mesenchymal stem cells could attenuate microglia-mediated neuroinflammation via the IRAK1/TRAF6 pathway and subsequent neural deficits following ischemic stroke [42]. TRAF6 is an upstream signal mediator of IKK*α*/*β* in the nuclear factor-kappa B (NF-*κ*B) signaling pathway [43]. NF-*κ*B used to be considered as a typical proinflammatory pathway [44]. Genetic evidence in mice has revealed the NF-*κ*B pathway has both pro- and anti-inflammatory roles [45]. These results showed that exosomes derived from cardiomyocytes deliver miR-146a-5p by targeting TRAF6 and may participate in macrophage polarization, demonstrating both pro- and anti-inflammatory roles.

## 5. Conclusion

In brief, this study showed that exosomal miR-146a-5p derived from cardiomyocytes could promote macrophage polarization and act both as a pro- and anti-inflammatory agent. Furthermore, exosomal miR-146a-5p promoted angiogenesis in macrophages. Exosomal miR-146a-5p may represent a potentially beneficial target for myocardial infarction treatment.

## Figures and Tables

**Table 1:** Clinical parameters of samples.

Sample	Sex	Age	Clinical symptoms	History of peripheral vascular disease	CKMB (U/L)	CK (U/L)	cTnI (ng/ml)	ECG	Range of lesions
MI 1	Male	60	Chest pain, shortness of breath	No	47.3	935	3.01	ST segment elevation	Three-vessel lesion
MI 2	Male	32	Chest pain	No	20	149	2.83	ST segment elevation	Single vessel lesion
MI 3	Male	38	Chest pain, heart failure	No	94.8	1791	2.88	ST segment elevation	Anterior descending branch + circumflex branch lesion
MI 4	Male	44	Chest pain	No	24.4	811	4.12	ST segment elevation	Single vessel lesion
MI 5	Male	50	Chest pain	No	33.5	594	1.79	ST segment elevation	Three-vessel lesion
NC 1	Male	51	Normal	No	8.3	141	0.005	Normal	No
NC 2	Male	48	Normal	No	9.6	91	<0.003	Normal	No
NC 3	Male	77	Normal	No	12.6	96	0.009	Normal	No
NC 4	Male	80	Normal	No	9.8	36	0.006	Normal	No
NC 5	Male	58	Normal	No	9.2	55	0.005	Normal	No

Note: MI: myocardial infarction; NC: negative control; CKMB: creatine kinase isoenzyme MB; CK: choline kinase; cTnI: cardiac troponin I; ECG: electrocardiogram.

**Table 2:** The information and dilution concentration of all the primary antibodies and secondary antibodies.

Name	Company	ID	Dilution concentration
TRAF6	Abcam	ab33915	1 : 2000
Phosphorylated IKK*α*/*β*	Cell Signaling Technology	2694	1 : 1000
IKK*β*	Cell Signaling Technology	8943	1 : 1000
GAPDH	Abcam	ab8245	1 : 2000
Anti-rabbit IgG antibody	Abcam	ab205718	1 : 5000

## Data Availability

The data used to support the findings of this study are included within the article.

## Supplementary Materials

Supplementary Materials Primer sequence information of genes.
