# Supplementary material for: Role of Cardiomyocyte-Derived Exosomal MicroRNA-146a-5p in Macrophage Polarization and Activation
Source: Dis Markers. 2022 May 2;2022:2948578. doi: 10.1155/2022/2948578 (PMC9085364; doi:10.1155/2022/2948578)
Supplement: Supplementary Materials — Primer sequence information of genes. [file 2948578.f1.docx]

Table S1 Primer sequence information of genes.

| **Gene** | | **ID** | **Primer sequence（5`-3`）** | | | | **Tm value** | | **CG%** | | **Lengths** |  |
| --- | --- | --- | --- | --- | --- | --- | --- | --- | --- | --- | --- | --- |
| GAPDH | | NM_017008.4 | sense | GCCAAGGTCATCCATGACAAC | | | 59.9 | | 52.4 | | 152 |  |
|  |  |  | antisense | GTGGATGCAGGGATGATGTTC | | | 59.8 | | 52.4 | |  |  |
| Arg1 | | NM_017134.3 | sense | ATTGGCAAAGTGATGGAAGAGAC | | | 59.24 | | 43.48 | | 287 |  |
|  |  |  | antisense | CAAGACAAGGTCAACGCCAC | | | 59.69 | | 55 | |  |  |
| IL4RA | | NM_133380.2 | sense | ACTTCTACGTGTGAGTGGCAGC | | | 59.5 | | 54.5 | | 154 |  |
|  |  |  | antisense | CCTCTATGGCCATTTGGCAC | | | 60.3 | | 55 | |  |  |
| IL10 | | NM_012854.2 | sense | GCAGGACTTTAAGGGTTACTTGG | | | 59.24 | | 47.83 | | 223 |  |
|  |  |  | antisense | ATCATTCTTCACCTGCTCCACT | | | 59.42 | | 45.45 | |  |  |
| TNFα | | NM_012675.3 | sense | CACCACGCTCTTCTGTCTACTG | | | 60.67 | | 54.55 | | 147 |  |
|  |  |  | antisense | GCTACGGGCTTGTCACTCG | | | 60.81 | | 63.16 | |  |  |
| iNOS | | NM_012611.3 | sense | AAACAACAGGAACCTACCAGCTC | | | 60.75 | | 47.83 | | 192 |  |
|  |  |  | antisense | CACTGTTAGTGGCGTAAAGTATGTG | | | 60.16 | | 44 | |  |  |
| VEGFA | | NM_001287107.1 | sense | AAAGCCCATGAAGTGGTGAAG | | | 59.2 | | 47.6 | | 255 |  |
|  |  |  | antisense | CATCTCTCCTATGTGCTGGCTTT | | | 60.5 | | 47.8 | |  |  |
| CCL2 | NM_031530.1 | | sense | | GGCCTGTTGTTCACAGTTGCT | 60 | | 52.4 | | 118 | | |
|  |  |  | antisense | | GCCGACTCATTGGGATCATC | 59.8 | | 55 | |  |  |  |
| IL-6 | NM_012589.2 | | sense | | TTCTCTCCGCAAGAGACTTCC | 58.7 | | 52.4 | | 116 | | |
|  |  |  | antisense | | GTGGGTGGTATCCTCTGTGAAG | 58.2 | | 54.5 | |  |  |  |
| IL1 | NM_017019.1 | | sense | | GATCAGCACCTCACAGCTTCC | 59.5 | | 57.1 | | 204 | | |
|  |  |  | antisense | | TAGAGTCGTCTCCTCCCGATG | 59.1 | | 57.1 | |  |  |  |
| CCL3 | NM_013025.2 | | sense | | GACTGCCTGCTGCTTCTCCTAT | 60.5 | | 54.5 | | 166 | | |
|  |  |  | antisense | | AGTGATGTATTCTTGGACCCAGG | 59.8 | | 47.8 | |  |  |  |
